# Multimorbidity and co-morbidity in atrial fibrillation and effects on survival: findings from UK Biobank cohort

**DOI:** 10.1093/europace/eux322

**Published:** 2017-11-02

**Authors:** Bhautesh Dinesh Jani, Barbara I Nicholl, Ross McQueenie, Derek T Connelly, Peter Hanlon, Katie I Gallacher, Duncan Lee, Frances S Mair

**Affiliations:** 1 General Practice and Primary Care, Institute of Health and Wellbeing, College of Medical, Veterinary and Life Sciences, University of Glasgow, Glasgow, UK; 2 Institute of Cardiovascular and Medical Sciences, College of Medical, Veterinary and Life Sciences, University of Glasgow, Glasgow, UK; 3 School of Mathematics and Statistics, University of Glasgow, Glasgow, UK

**Keywords:** Atrial fibrillation, Co-morbidity, All-cause mortality, UK Biobank

## Abstract

**Aims:**

To examine the number and type of co-morbid long-term health conditions (LTCs) and their associations with all-cause mortality in an atrial fibrillation (AF) population.

**Methods and results:**

Community cohort participants (UK Biobank *n *= 502 637) aged 37–73 years were recruited between 2006 and 2010. Self-reported LTCs (*n *= 42) identified in people with AF at baseline. All-cause mortality was available for a median follow-up of 7 years (interquartile range 76–93 months). Hazard ratios (HRs) examined associations between number and type of co-morbid LTC and all-cause mortality, adjusting for age, sex, socio-economic status, smoking, and anticoagulation status. Three thousand six hundred fifty-one participants (0.7% of the study population) reported AF; mean age was 61.9 years. The all-cause mortality rate was 6.7% (248 participants) at 7 years. Atrial fibrillation participants with ≥4 co-morbidities had a six-fold higher risk of mortality compared to participants without any LTC. Co-morbid heart failure was associated with higher risk of mortality [HR 2.96, 95% confidence interval (CI) 1.83–4.80], whereas the presence of co-morbid stroke did not have a significant association. Among non-cardiometabolic conditions, presence of chronic obstructive pulmonary disease (HR 3.31, 95% CI 2.14–5.11) and osteoporosis (HR 3.13, 95% CI 1.63–6.01) was associated with a higher risk of mortality.

**Conclusion:**

Survival in middle-aged to older individuals with self-reported AF is strongly correlated with level of multimorbidity. This group should be targeted for interventions to optimize their management, which in turn may potentially reduce the impact of their co-morbidities on survival. Future AF clinical guidelines need to place greater emphasis on the issue of co-morbidity.

**What’s new?**

- Survival in middle-aged individuals with self-reported atrial fibrillation was strongly correlated with the level of multimorbidity.
- Presence of certain non-cardiometabolic conditions, such as chronic obstructive pulmonary disease and osteoporosis, was associated with a significantly higher risk of mortality, and the strength of these associations was similar to those observed with co-morbid cardiometabolic conditions such as heart failure.
- Atrial fibrillation patients with co-morbidities should be targeted for interventions to optimize their management, which in turn may potentially reduce the impact of their co-morbidities on survival.

## Introduction

Atrial fibrillation (AF) is the commonest sustained arrhythmia with a worldwide prevalence estimated to be more than 33 million people.^1^ The prevalence of AF is increasing globally, partly attributable to improved surveillance and the increasing prevalence of lifestyle-related cardiovascular disease risk factors. The presence of AF is associated with a nearly two-fold increase in the risk of all-cause mortality.^2^ Recently, death has been reported as the most frequent major adverse clinical event among AF patients, with significantly higher event rates than stroke or systemic embolism.^3^

Multimorbidity, defined as the presence of two or more long-term conditions (LTC),^4^ or co-morbidity, defined as the presence of one more LTC in addition to an index condition,^5^ is very common in people with AF.^6–8^ A few studies have investigated co-morbidity in AF, with some variations in the sample size, LTCs considered, and the observed findings. A large Swedish study of 272 186 patients with incidental AF reported 69.5% prevalence of at least one other co-morbid condition as compared to 29.2% in matched controls (considered seven LTCs based on records from a national patient register).^6^ Similarly, a study in Belgium reported significantly more co-morbidities among 1830 AF patients when compared with the controls (considered 14 LTCs based on general practice records).^8^ A study based in the USA using the Rochester Epidemiology Project (REP) database found higher prevalence of most co-morbid conditions in 1430 AF patients compared with their matched controls (considered 19 LTCs based on hospital records).^7^

The association of co-morbidity with all-cause mortality in the AF population has been studied to some extent previously.^6^^,^^7^^,^^9–11^ However, there are significant gaps in the existing knowledge on the patterns of prevalence of co-morbidity in AF and any impact on mortality. Most of the published studies have examined a limited number of co-morbidities that included mostly cardiometabolic or chronic obstructive pulmonary disease (COPD),^6^^,^^9–11^ with the exception of the REP database study.^7^ Therefore, the effects, if any, of a range of non-cardiometabolic co-morbid conditions on all-cause mortality remain largely unknown. Furthermore, the relationship of multimorbidity with all-cause mortality in people with AF remains unclear.

The aims of this study were as follows:

1. To compare the number and type of co-morbid LTCs and their association with all-cause mortality in participants with and without self-reported AF;
2. To compare the effect of the presence of different co-morbid conditions on the risk of all-cause mortality in participants with self-reported AF.

## Methods

### Study design and data collection

UK Biobank is a large prospective cohort of 502 640 participants (age 37–73 years) recruited between 2006 and 2010. The participants attended 1 of 22 assessment centres across England, Scotland, and Wales and completed a touch screen and nurse-led questionnaire with information on demographics, health-related behaviour, and self-reported LTCs at the time of recruitment. The participants also gave consent for prospective data linkage to national mortality data. Participants provided full informed consent to participate in UK Biobank, and this study was covered by the generic ethics approval for UK Biobank studies from the NHS National Research Ethics Service (approval letter dated 17 June 2011, Ref 11/NW/0382).

### Description of clinical variables and outcomes

Age was categorized into >65 years and <65 years (based on CHA_2_DS_2_-VASc cut-off), sex was used as a categorical variable, and socio-economic status was classified into quintiles based on Townsend score (a measure of deprivation in the UK); Category 1 was the least deprived, and Category 5 was the most deprived category (https://census.ukdataservice.ac.uk/get-data/related/deprivation). Smoking status was classified into two categories: non-smokers and previous/current smokers. The list of 42 self-reported LTCs was used based on the previously published literature on multimorbidity (Supplementary material online, *Table S1*).^4^^,^^12^ Chronic pain was defined as present if the participant reported pain that had persisted for more than 3 months in at least one of the seven body sites listed or ‘all over the body’; a group of 20 LTCs known to have pain as one of the major symptoms were labelled as ‘painful conditions’ (see Supplementary material online, *Table S1*).^12^ Presence of depressive symptoms was defined based on the results of high Patient Health Questionnaire (PHQ-2), a score of 2 or more was used as a cut-off.^13^ Multimorbidity was classified on the basis of count into three categories: presence of no other LTCs, presence of 1–3 LTCs, and presence of 4 or more LTCs. Multimorbidity was also categorized as cardiometabolic or non-cardiometabolic, the latter including conditions used in the CHA_2_DS_2_-VASc score. All-cause mortality was recorded by data linkage with national UK mortality registers.

### Statistical methods

The initial analysis involved using the data from the whole UK Biobank cohort and was split into two stages via the following analysis plan. In the first stage, we used data on individuals with and without self-reported AF, and the aim was to quantify the risk of all-cause mortality between participants with and without self-reported AF. In this stage, demographic information, smoking status, number, and type of co-morbid health conditions were compared between participants with and without self-reported AF using the χ^2^ tests for categorical variables and the unpaired Student’s *t*-tests for continuous variables. Cox’s proportional hazards multivariable regression analysis was used to compare the all-cause mortality in different participant categories based on presence of self-reported AF and the number of other LTCs. Participants with no LTCs were used as the reference category, and five other categories were created for comparison as described above. The models fitted were adjusted for age, sex, Townsend quintile, and smoking status. Participants with missing values were excluded from the regression modelling. In building the Cox models, all covariates were initially included in the model, and any non-significant effects (at the 5% level) were removed one by one until only significant variables remained. The results of this analysis were presented as hazard ratios (HRs) and 95% confidence intervals (CI).

The second stage of the analysis was performed using data from participants with self-reported AF only, and the aim was to compare the effect of the presence of different co-morbid conditions on the risk of all-cause mortality. In this analysis, a survival plot was used to compare cumulative survival between self-reported AF participants with different numbers of co-morbid LTCs (none, 1–3, 4 or more). A Cox’s proportional hazards multivariable regression model was constructed using all-cause mortality as outcome variable among participants with AF. Presence of any other cardiometabolic LTC and presence of any other non-cardiometabolic LTC were used as predictor variables in the same model. As before, the models were adjusted for age, sex, Townsend quintile, and smoking status. In addition, the regression models performed using participants with AF were also adjusted for their anticoagulation status at baseline (on warfarin—yes or no). As with the Stage 1 analysis, the model-building process initially included all variables in the model, and any non-significant effects (at the 5% level) were then removed one by one until only significant variables remained. The results were presented with HRs and 95% CI. The final part of this Stage 2 analysis was to construct individual Cox’s proportional hazards multivariable regression models to study the adjusted effect size for the presence of each individual LTC (excluding those with a prevalence of < 1% among participants with AF) on all-cause mortality. Finally, a forest plot was constructed demonstrating the adjusted HRs for all-cause mortality for co-morbid individual LTCs among participants with self-reported AF; for each cardiometabolic and selected non-cardiometabolic condition (with a statistically significant association). All missing values were excluded in all of the regression models in Stage 2. The results are presented in detail in a table in the supplementary section (Supplementary material online, *Table S2*).

In addition, the regression models described above were repeated using age as a continuous variable for sensitivity analysis. These results are also presented in the supplementary section (Supplementary material online, *Tables S3* and *S4*).

All statistical analyses were done using the R software (version 3.2.2).

## Results

### Participant characteristics

In total, 502 640 UK Biobank participants were included in the analyses, whereas 3 participants were excluded due to unavailability of information on all-cause mortality. At the time of recruitment, 3651 (0.7%) participants who enrolled in the UK Biobank reported having AF. The mean age of participants was 61.9 years, and the range was 40–70 years. *Table **1* compares the demographic characteristics, smoking status, distribution of co-morbidity, and anticoagulation status of participants with and without self-reported AF in the UK Biobank at baseline. Participants with self-reported AF were significantly older, more likely to be male, and more likely to be smokers compared with participants without self-reported AF.

**Table 1:** Participants with and without self-reported AF

	Participants with self-reported AF (*n* = 3651)	Participants without self-reported AF (*n* = 498 986)	*P*-value
Demographics			
Age (years), mean (SD)	61.9 (6)	56.4 (8)	<0.001
Age 65–73 (years)	1201/3651 (32.9%)	72 687 (14.5%)	<0.001
Female-sex	1159/3651 (31.7%)	272 306 (54.5%)	<0.001
Townsend score			
1	767/3648 (21%)	99 919/498 362 (20.1%)	<0.001
2	783/3648 (21.4%)	99 335/498 362 (19.9%)	
3	744/3648 (20.3%)	99 668/498 362 (20%)	
4	733/3648 (20%)	99 662/498 362 (20%)	
5	621/3648 (17.1%)	99 778/498 362 (20%)	
Smoking—current/previous (vs. never smoked)	1833/3625 (50.5%)	224 252/496 060 (45.2%)	<0.001
Distribution of multimorbidity			
MM count—mean	1.74	1.19	<0.001
Number of co-morbid conditions			
None	713/3641 (19.6%)	172 599/497 151 (34.7%)	<0.001
1–3	2524/3641 (69.3%)	299 334/497 151 (60.2%)	
≥4	404/3641 (11.1%)	25 218/497 151 (5.1%)	
Presence of co-morbid cardiometabolic conditions (included in CHADS_2_VASC)			
Presence of at least one cardiometabolic condition	2013/3641 (55.2%)	153 214/497 151 (30.8%)	<0.001
Hypertension	1630/3641 (44.8%)	131 694/497 151 (26.5%)	<0.001
Diabetes	331/3641 (9.1%)	25 172/497 151 (5%)	<0.001
Coronary heart disease	430/3641 (11.8%)	22 301/497 151 (4.5%)	<0.001
Stroke/TIA	258/3641 (7.1%)	8596/497 151 (1.7%)	<0.001
Heart failure	97/3641 (2.7%)	706/497 151 (0.14%)	<0.001
Peripheral vascular disease	13/3641 (0.4%)	1266/497 151 (0.25%)	0.22
Presence of co-morbid non-cardiometabolic conditions			
Presence of at least one non-cardiometabolic condition	2173/3641 (59.7%)	260 418/497 151 (52.4%)	<0.001
Chronic pain symptoms^a^	1732/3640 (47.6%)	216 988/496 804 (43.6%)	<0.001
Painful condition^a^	865/3641 (23.7%)	83 127/497 151 (16.7%)	<0.001
Depressive symptoms (based on PHQ-2>1)	535/3426 (15.6%)	80 893/464 097 17.4%)	0.005
Asthma	426/3641 (11.7%)	57 867/497 151 (11.6%)	0.89
Dyspepsia	401/3641 (10.9%)	38 675/497 151 (7.7%)	<0.001
Cancer	347/3641 (9.5%)	38 276/497 151 (7.7%)	<0.001
Thyroid disorders	301/3641 (8.2%)	28 839/497 151 (5.7%)	<0.001
Prostate disorders	153/3641 (4.2%)	8107/497 151 (1.7%)	<0.001
Psoriasis/eczema	129/3641 (3.5%)	17 707/497 151 (3.5%)	0.96
Connective tissue disorders^a^	101/3641 (2.7%)	10 932/497 151 (2.1%)	0.018
COPD	99/3641 (2.7%)	8218/497 151 (1.6%)	<0.001
Irritable bowel syndrome	77/3641 (2.1%)	11 417/497 151 (2.2%)	0.47
Osteoporosis	74/3641 (2.0%)	7968/497 151 (1.6%)	0.03
Diverticular disease	67/3641 (1.8%)	5338/497 151 (1.1%)	0.07
Migraine	66/3641 (1.8%)	14 324/497 151 (2.9%)	<0.01
Glaucoma	64/3641 (1.7%)	5252/497 151 (1.1%)	0.03
Anxiety	60/3641 (1.6%)	8966/497 151 (1.8%)	0.48
Inflammatory bowel disease	31/3641 (0.8%)	4200/497 151 (0.8%)	0.96
Epilepsy	29/3641 (0.8%)	4027/497 151 (0.8%)	0.93
Treated constipation	5/3641 (0.1%)	398/497 151 (0.08%)	0.22
Chronic kidney disease	24/3641 (0.6%)	1287/497 151 (0.25%)	<0.01
Chronic sinusitis	19/3641 (0.5%)	3084/497 151 (0.6%)	0.45
Bronchiectasis	18/3641 (0.5%)	1117/497 151 (0.2%)	<0.01
Meniere‘s disease	17/3641 (0.5%)	1359/497 151 (0.3%)	0.02
Endometriosis	17/3641 (0.5%)	4045/497 151 (0.8%)	0.02
Pernicious anaemia	16/3641 (0.4%)	1501/497 151 (0.3%)	0.13
Viral hepatitis	15/3641 (0.4%)	1322/497 151 (0.3%)	0.08
Chronic liver disease	11/3641 (0.3%)	9600/497 151 (1.9%)	0.13
Chronic fatigue syndrome	9/3641 (0.2%)	3642/497 151 (0.7%)	0.08
Alcohol problems	8/3641 (0.2%)	803/497 151 (0.2%)	0.38
Schizophrenia/bipolar disorder	8/3641 (0.2%)	1989/497 151 (0.4%)	0.08
Parkinson’s disease	7/3641 (0.2%)	851/497 151 (0.2%)	0.75
Multiple sclerosis	5/3641 (0.1%)	1772/497 151 (0.5%)	0.02
Other psychoactive substance abuse	1/3641 (0.03%)	97/497 151 (0.02%)	0.73
Dementia	1/3641 (0.03%)	123/497 151 (0.03%)	0.91
Polycystic ovary	1/3641 (0.03%)	622/497 151 (0.1%)	0.09
Anorexia or bulimia	0	370/497 151 (0.07%)	0.09

*n* = 502 637.

AF, atrial fibrillation; COPD, chronic obstructive pulmonary disease; MM, multimorbidity; PHQ-2, Patient Health Questionnaire; TIA, transient ischaemic attack.

a Chronic pain symptoms: pain present for more than 3 months; painful conditions: back pain, joint pain, headaches (not migraine), sciatica, plantar fasciitis, carpal tunnel syndrome, fibromyalgia, arthritis, shingles, disc problem, prolapsed disc/slipped disc, spine arthritis/spondylitis, ankylosing spondylitis, back problem, osteoarthritis, gout, cervical spondylosis, trigeminal neuralgia, disc degeneration, and trapped nerve/compressed nerve; connective tissue disorders: myositis/myopathy, systemic lupus erythematosus, Sjogren’s syndrome/sicca syndrome, dermatopolymyositis, dermatomyositis, polymyositis, scleroderma, systemic sclerosis, rheumatoid arthritis, psoriatic arthropathy, polymyalgia rheumatica, and malabsorption syndrome/coeliac disease.

### Distribution of co-morbidity in participants with and without atrial fibrillation

Approximately 80% of participants with self-reported AF had at least one other co-morbid LTC compared to nearly 65% among participants without self-reported AF. The majority of participants with self-reported AF (55.2%) had at least one other cardiometabolic condition compared to 30.8% participants without self-reported AF. Approximately 45% of AF participants were on anticoagulants compared with 0.7% of participants without self-reported AF. Participants with self-reported AF were more likely to have chronic pain symptoms (47.6%) and co-morbid painful condition (23.7%) as compared to participants without AF (43.6% and 16.7%, respectively).

### Multimorbidity and survival in participants with and without atrial fibrillation

The median duration for follow-up was 7 years (interquartile range 76–93 months). Two hundred and forty-eight (6.7%) participants with AF had died at the end of the follow-up period as compared to 14 171 (2.8%) participants without self-reported AF. Vascular deaths were reported in 105 (2.5%) participants with self-reported AF compared with 2895 (0.6%) participants without AF. *Table **2* compares the relationship between all-cause mortality and the number of LTCs reported at baseline, among participants with and without self-reported AF. The results were adjusted for age, sex, smoking, and socio-economic status. Participants with self-reported AF had 107% higher risk of mortality compared with participants in the reference category with no LTCs. Participants with self-reported AF and four or more other co-morbid conditions had nearly six-fold higher risk of mortality as compared to the reference group.

**Table 2:** Relationship of multimorbidity with all-cause mortality in participants with and without self-reported AF using multivariate Cox’s proportional hazards regression analysis

Predictor variables	Hazard ratios (95% CI)	*P*-value	Regression coefficients
Age > 65 (against age < 65 as reference)	2.31 (2.23–2.40)	<0.001	0.84
Sex—male (against female as reference)	1.72 (1.66–1.78)	<0.001	0.54
Townsend score categories			
Category 1: least deprived (reference)	1		
Category 2	1.00 (0.94–1.05)	0.94	0.001
Category 3	1.08 (1.02–1.14)	0.001	0.081
Category 4	1.19 (1.13–1.26)	<0.001	0.18
Category 5: most deprived	1.46 (1.39–1.54)	<0.001	0.38
Smoking—current/previous (vs. never smoked as reference)	1.67 (1.61–1.73)	<0.001	0.51
Participants classified into different groups based on presence of AF and the number of long-term conditions			
Participants without any long-term conditions (LTCs) (reference)	1		
AF present and no other LTCs	2.06 (1.40–3.04)	<0.001	0.72
AF absent and 1–3 LTCs	2.07 (1.98–2.16)	<0.001	0.73
AF present and 1–3 other LTCs	3.17 (2.70–3.73)	<0.001	1.15
AF absent and 4 or more LTCs	4.25 (4.00–4.52)	<0.001	1.44
AF present and 4 or more other LTCs	6.80 (5.23–8.83)	<0.001	1.91
Concordance (measure of model performance) = 0.70			

*n* = 502 637. UK Biobank participants (5261 excluded due to missing values); number of deaths = 14 206.

AF, atrial fibrillation; CI, confidence intervals; Outcome, all-cause mortality at 7 years.

### Distribution of co-morbidity and survival in participants with atrial fibrillation

**Figure 1:**
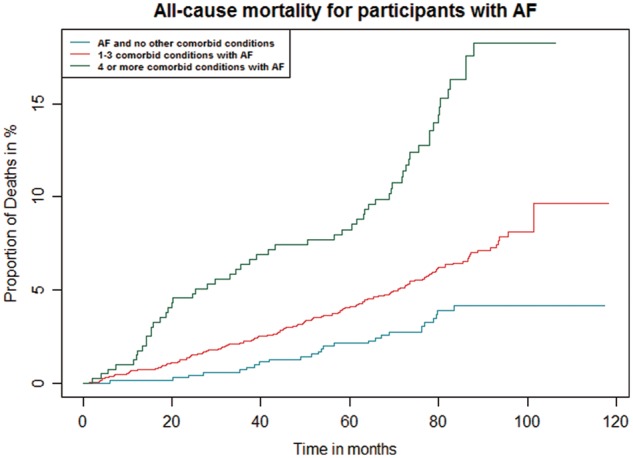
Cumulative survival plot showing probability of all-cause mortality among self-reported AF participants with different levels of multimorbidity. *n* = 3651; AF participants (107 excluded due to missing values).

*Figure* *1* compares the unadjusted survival among participants with AF on the basis of number of associated co-morbid conditions. Nearly one in five AF participants with four or more other co-morbid conditions had died at the end of 7 years of follow-up, compared with approximately 1 in 20 AF participants with no co-morbid conditions.

*Table* *3* shows the adjusted effect sizes for the presence of type of co-morbid conditions (cardiometabolic and non-cardiometabolic) on all-cause mortality in the AF population. The presence of a single cardiometabolic condition (CHA2DS2-VASc conditions) was associated with an 83% higher risk of mortality, whereas the presence of any single non-cardiometabolic condition was associated with a 45% higher risk of mortality.

**Table 3:** Relationship between presence of cardiometabolic and non-cardiometabolic co-morbidity and all-cause mortality in AF participants using multivariate Cox’s proportional hazards model

Predictor variables	Hazard ratios (95% CI)	*P*-value	Regression coefficients
Age > 65 (against age < 65 as reference)	1.47 (1.13–1.90)	<0.001	0.38
Sex-male (against female as reference)	2.14 (1.54–2.98)	<0.001	0.76
Townsend score categories			
Category 1: least deprived (reference)	1		
Category 2	1.45 (0.92–2.28)	0.10	0.37
Category 3	1.89 (1.22–2.92)	<0.001	0.63
Category 4	1.73 (1.11–2.69)	0.01	0.55
Category 5: most deprived	2.03 (1.30–3.16)	<0.001	0.71
Smoking—current/previous (against never smoked as reference)	1.28 (0.98–1.67)	0.065	0.24
Anticoagulation status—on warfarin (against not on anti-coagulants as reference)	1.34 (1.02–1.75)	0.031	0.29
Presence of at least one other cardiometabolic condition (against no co-morbid cardiometabolic condition as reference)	1.83 (1.36–2.45)	<0.001	0.60
Presence of at least one other non-cardiometabolic condition (against no co-morbid non-cardiometabolic condition)	1.45 (1.11–1.91)	0.006	0.37
Concordance (measure of model performance) = 0.68			

*n* = 3651 participants with AF (107 excluded due to missing values); number of deaths = 241.

AF, atrial fibrillation; CI, confidence interval; Outcome, all-cause mortality at 7 years.

**Figure 2:**
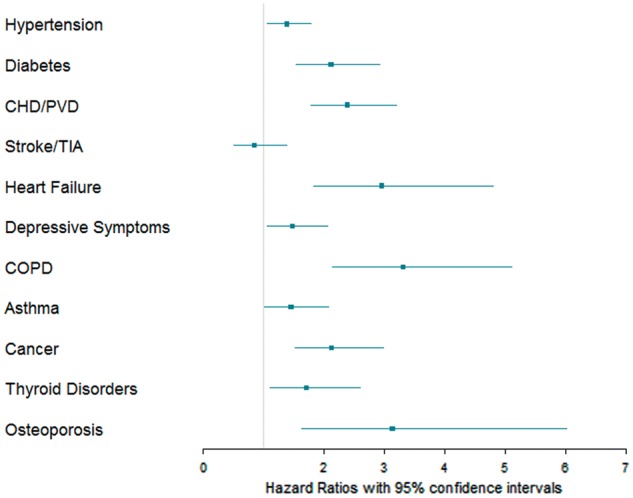
Forest plot of hazard ratio for the presence of different cardiometabolic and non-cardiometabolic conditions and all-cause mortality in participants with AF. *n* = 3651 (97 excluded due to missing values). Depressive symptoms include PHQ-2 ≥2 Results adjusted for age, sex, socio-economic, smoking, and anticoagulation status. CHD, coronary heart disease; PHQ, Patient Health Questionnaire; PVD, peripheral vascular disease.

*Figure* *2* compares the adjusted effect sizes for the presence of individual cardiometabolic and non-cardiometabolic conditions on survival among AF participants. Notably, presence of co-morbid heart failure was associated with the highest risk of mortality (HR 2.96, 95% CI 1.83–4.80) among cardiometabolic conditions, whereas previous history of stroke or transient ischaemic attack (TIA) co-morbid with AF was not associated with higher risk of mortality (HR 0.85, 95% CI 0.52–1.39). Among non-cardiometabolic conditions, notably, presence of COPD (HR 3.31, 95% CI 2.14–5.11), osteoporosis (HR 3.13, 95% CI 1.63–6.01), and cancer (HR 2.13, 95% CI 1.53–2.98) co-morbid with AF were associated with significantly higher risk of mortality compared with AF participants without these conditions, respectively (*Figure **2*). Among AF participants, the adjusted effect size for the presence of co-morbid COPD and osteoporosis on mortality was similar to the effect sizes observed for any cardiometabolic condition. These results are presented in detail in Supplementary material online, *Table S2*. The observed trend in results was unchanged in the sensitivity analysis using age as a continuous variable (see Supplementary material online, *Table S3* and *S4*).

## Discussion

### Summary of findings

Multimorbidity was common among middle-aged and older participants, aged 40–70 years, recruited from a community setting with self-reported AF. The majority of AF participants reported the presence of at least one other co-morbid LTC with both cardiometabolic and non-cardiometabolic co-morbidities prevalent in approximately 80% of the participants. At the end of the 7 years of follow-up period, a dose–response association was observed between level of multimorbidity and all-cause mortality with an increase in the number of co-morbid LTCs alongside self-reported AF being associated with lower chances of survival. Presence of all individual cardiometabolic conditions was associated with a significantly higher risk of mortality among AF participants, with the exception of stroke/TIA. Presence of certain non-cardiometabolic conditions was also associated with a significantly higher risk of mortality, notably the risk of mortality associated with co-morbid COPD and osteoporosis was similar to the effect sizes observed for any cardiometabolic condition. These results were adjusted for age, sex, socio-economic status, smoking status, and anticoagulant status at baseline.

### Comparison of findings to other studies and implications

The prevalence of co-morbidity observed in our study was similar to those previously reported for people with AF. There are some key similarities and differences between the findings of our study and other studies examining the effects of co-morbidity on all-cause mortality in AF population.^6^^,^^7^^,^^9–11^ All of the previous studies report a statistically significant higher risk of mortality with co-morbid COPD among AF participants; however, the effect sizes were smaller than this study.^6^^,^^9–11^ In a much larger Swedish study, the effect sizes for the presence of certain concomitant conditions on mortality were evaluated in participants with AF in three different age groups.^6^ Among cardiometabolic conditions, co-morbid heart failure was associated with the highest risk of mortality in AF participants <65 years with a similar effect size as reported here.^6^ Similar to the findings in our study, presence of cancer was also associated with a high risk of mortality.^6^ The American study using the REP database reported no statistically significant association for the presence of co-morbid COPD, osteoporosis, and asthma with all-cause mortality among participants with AF.^7^ However, these studies did not adjust for smoking, socio-economic, and anticoagulation status at baseline which are important potential confounding variables.^6^^,^^7^ In the ROCKET AF trial, various cardiometabolic conditions (such as stroke, diabetes, and heart failure) and COPD were found to have a statistically significant association with all-cause mortality; however, the list of conditions examined was not as comprehensive as our study.^14^ The ORBIT-AF study reported higher all-cause mortality for AF patients with co-morbid cancer but only studied the effect of cancer.^15^ Similarly, other studies have reported a significant association of mainly cardiometabolic conditions co-morbid with AF, but they have not examined the effects of non-cardiometabolic conditions.^16^^,^^17^ In summary, the effects of non-cardiometabolic conditions have not been studied comprehensively before, and most of the previous studies have not adjusted for the effects of a wide range of confounding factors in their models. Importantly, the distribution of multimorbidity among AF patients and its relationship with mortality has not been studied before.

There are a number of implications of our findings. Co-morbidity and multimorbidity should be taken into account in risk stratification of patients with AF. The current guidelines for risk stratification in AF patients is mainly based on determining the need for anticoagulation for prevention of thromboembolism,^18^ which in turn is largely based on using the CHADS_2_VASC score which only takes cardiometabolic co-morbidity into account. The current guidelines for management of AF do not consider the risk of mortality in risk stratification. Recent evidence has shown that the risk of mortality is much higher for patients with newly diagnosed AF as compared to the risk of stroke^3^^,^^19^ and that patients are more likely to die of a cardiovascular event, usually heart failure.^20^ Importantly, the degree of multimorbidity as well as the co-morbidity of non-cardiometabolic conditions, such as COPD and osteoporosis, may have to be considered when calculating the risk of mortality in patients with AF. Further research in this area needs to focus on different causes of mortality and other clinical outcomes in the AF population, such as hospitalization, and the role of co-morbid conditions in these relationships. Equally, exploration of interventions that may help enhance patient capacity to manage their chronic illnesses and multimorbidity are merited, as there is emerging evidence that complex interventions focusing on improving patient capacity for self-care may be effective in reducing hospital admission in patients with multiple LTCs.^21^ Finally, both cardiometabolic and non-cardiometabolic co-morbidities was very common among AF participants. This may have important implications for polypharmacy and drug interactions. For example, nearly half of the participants with AF in this sample reported having chronic pain symptoms. This puts them at risk of potentially dangerous prescribing such as the co-prescribing of non-steroidal anti-inflammatory agents and anticoagulants.

### Strengths

This study has certain key strengths such as a large sample size, recruitment from different areas of the UK, good data completion rates, and adjustment for multiple confounding factors.

### Limitations

There are a few important limitations of our findings. First, all LTCs including AF were self-reported and the diagnosis could not be verified from clinical notes. Secondly, participants with more LTCs are likely to have more health care utilization, which in turn may potentially increase the probability of detecting silent AF. In addition, information on the duration and the type of AF (paroxysmal or persistent) was not known, which may have influenced the risk of mortality. In future, it may be possible to get information on Holter monitoring for UK Biobank participants, which may help with better categorization and validation of the self-reported AF cohort. The observed effect sizes for LTCs had large CIs which could be attributed to the relatively smaller sample size. It is possible that some participants in the cohort may have other undiagnosed conditions that may potentially influence the observed trends in the results.^22^ Finally, the UK Biobank population has a higher proportion of Caucasians and affluent people participating compared with the UK general population.

## Conclusions

Multimorbidity was highly prevalent in this middle-aged to older cohort of patients with AF. Importantly, those with multimorbidity are at increased risk of death over a follow-up period of 5–10 years. These findings suggest that such patients should be prioritized for interventions to optimize their management and in some cases to adjust their lifestyle to reduce the impact of their co-morbidities on survival. There may be a small number of multimorbid and frail patients with AF, likely to have poor life expectancy, who should be offered conservative and less invasive treatment options. However, it is possible that, in the past, patients with multimorbidity may have been denied effective treatments that might have improved their outcome, and we would suggest that in most cases effective treatments and interventions should not be withheld from these high-risk individuals. The problem of co-morbidity and multimorbidity needs to be given greater consideration in future AF guidelines.

## Supplementary Material

Supplementary Table

Supplementary Material.
