# Supplementary material for: Multimorbidity and co-morbidity in atrial fibrillation and effects on survival: findings from UK Biobank cohort
Source: Europace. 2017 Nov 2;20(FI 3):f329–36. doi: 10.1093/europace/eux322 (PMC6277149; doi:10.1093/europace/eux322)
Supplement: Supplementary Table [file eux322_supplementary_table.docx]

Title: Comorbidity in Atrial Fibrillation and Effects on Survival: Findings from UK Biobank Cohort

Supplementary Material

**Table S1 List of self-reported long-term conditions considered for multimorbidity count**

| Long term condition grouping | Conditions included as reported by participants |
| --- | --- |
| 1. Painful conditions | Back pain  Joint pain  Back pain  Joint pain  Headaches (not migraine)  Sciatica  Plantar fasciitis  Carpal tunnel syndrome  Fibromyalgia  Arthritis  Shingles  Disc problem  Prolapsed disc/slipped disc  Spine arthritis/spondylitis  Ankylosing spondylitis  Back problem  Osteoarthritis  Gout  Cervical spondylosis  Trigeminal neuralgia  Disc degeneration  Trapped nerve/compressed nerve |
| 1. Hypertension | Hypertension  Essential Hypertension |
| 1. Depression | Depression  Postnatal Depression |
| 1. Asthma | Asthma |
| 1. Coronary Heart Disease | Heart attack/Myocardial Infarction  Angina |
| 1. Dyspepsia | Gastro-oesophageal reflux (GORD)/gastric reflux  Oesophagitis /Barrett's oesophagus  Gastric stomach ulcers  Gastric erosions/gastritis  Duodenal ulcer  Dyspepsia/indigestion  Hiatus hernia  Helicobacter pylori |
| 1. Diabetes | Diabetic nephropathy  Diabetic neuropathy/ulcers  Diabetes  Type 1 diabetes  Type 2 diabetes  Diabetic eye disease |
| \| 1. Thyroid disorders \| \| --- \| | Thyroid problem (not cancer)  Hyperthyroidism/thyrotoxicosis  Hypothyroidism/myxoedema  Grave’s disease  Thyroid goitre  Thyroiditis |
| 1. Connective tissue disorders | Myositis/myopathy  Systemic Lupus Erythematosus  Connective tissue disorder  Sjogrens syndrome/sicca syndrome  Dermatopolymyositis  Scleroderma/systemic sclerosis  Rheumatoid arthritis  Psoriatic arthropathy  Dermatomyositis  Polymyositis  Polymyalgia Rheumatica  Malabsorption/coeliac disease |
| 1. Chronic Obstructive Pulmonary Disease (COPD) | COPD/chronic obstructive airways disease  Emphysema/chronic bronchitis  Emphysema |
| 1. Anxiety | Anxiety/panic attacks  Nervous breakdown  Post-traumatic stress disorder  Obsessive compulsive disorder  Stress  Insomnia  Psychological/psychiatric problem |
| 1. Irritable bowel syndrome | Irritable bowel syndrome |
| 1. Alcohol problems | Alcohol dependency  Alcoholic liver disease/alcoholic cirrhosis |
| 1. Other psychoactive substance abuse | Opioid dependency  Other substance abuse/dependency |
| 1. Treated constipation | Constipation |
| 1. Stroke/Transient Ischaemic Attack (TIA) | Stroke  TIA  Subarachnoid haemorrhage  Brain haemorrhage  Ischaemic stroke |
| 1. Chronic kidney disease | Polycystic kidney  Diabetic nephropathy  Renal/kidney failure  Renal failure requiring dialysis  Renal failure not requiring dialysis  Kidney nephropathy  Immunoglobulin A (IgA) nephropathy |
| 1. Diverticular disease | Diverticular disease  Diverticulitis |
| 1. Peripheral vascular disease | Peripheral vascular disease  Leg claudication/intermittent claudication |
| 1. Heart failure | Cardiomyopathy  Hypertrophic cardiomyopathy  Heart failure/pulmonary oedema |
| 1. Prostate disorders | Prostate problem (not cancer)  Enlarged prostate  Benign prostatic hypertrophy |
| 1. Glaucoma | Glaucoma |
| 1. Epilepsy | Epilepsy |
| 1. Dementia | Dementia  Alzheimer’s disease  Cognitive impairment |
| 1. Schizophrenia/bipolar disorder | Schizophrenia  Mania/  Bipolar disorder  Manic depression |
| 1. Psoriasis/eczema | Eczema  Dermatitis  Psoriasis |
| 1. Inflammatory Bowel Disease | Inflammatory Bowel Disease  Crohn’s disease  Ulcerative colitis |
| 1. Migraine | Migraine |
| 1. Chronic sinusitis | Chronic sinusitis |
| 1. Anorexia or bulimia | Anorexia  Bulimia  Other eating disorders |
| 1. Bronchiectasis | Bronchiectasis |
| 1. Parkinson’s disease | Parkinson’s disease |
| 1. Multiple Sclerosis | Multiple Sclerosis |
| 1. Viral Hepatitis | Infective/viral hepatitis  Hepatitis B  Hepatitis C  Hepatitis D  Hepatitis E |
| 1. Chronic Liver disease | Oesophageal varices  Non infective hepatitis  Liver failure/cirrhosis  Primary biliary cirrhosis |
| 1. Osteoporosis | Osteoporosis |
| 1. Chronic fatigue syndrome | Chronic fatigue syndrome |
| 1. Endometriosis | Endometriosis |
| 1. Meniere’s disease | Meniere’s disease |
| 1. Pernicious Anaemia | Pernicious Anaemia |
| 1. Polycystic ovary | Polycystic ovary |
| 1. Cancer | Lifetime diagnosis |

**Table S2 Hazard Ratio for presence of different cardiometabolic and non-cardiometabolic conditions and all-cause mortality in participants with and without AF**

|  | **Participants with AF**  **Hazard Ratios with 95% CI; p-value*** | **Participants without AF**  **Hazard Ratios with 95% CI; p-value**** |
| --- | --- | --- |
| **Presence of comorbid cardiometabolic conditions (included in CHADS2VASC)** | |  |
| **Hypertension** | **1.39 (1.07-1.79); 0.01** | **1.51 (1.46-1.56); <0.01** |
| **Vascular Disease (CHD or PVD)** | **2.39 (1.79-3.20); <0.01** | **2.12 (2.02-2.23); <0.01** |
| **Diabetes** | **2.12 (1.54-2.92); <0.01** | **2.14 (2.04-2.25); <0.01** |
| Stroke/TIA | 0.85 (0.52-1.39);0.53 | **2.24 (2.08-2.42); <0.01** |
| **Heart Failure** | **2.96 (1.83-4.80);<0.01** | **3.45 (2.78-4.27); <0.01** |
| **Presence of comorbid non cardiometabolic conditions** | |  |
| Chronic Pain Symptoms* | 0.93 (0.72-1.20); 0.58 | **1.17 (1.13-1.21); <0.01** |
| Painful Condition* | 1.09 (0.81-1.45); 0.55 | **1.12 (1.08-1.17); <0.01** |
| **Depressive Symptoms (based on PHQ-2>1)** | **1.48 (1.07-2.06); 0.01** | **1.33 (1.27-1.39); <0.01** |
| **Asthma** | **1.46 (1.02-2.08); 0.03** | **1.11 (1.05-1.17); <0.01** |
| Dyspepsia | 1.18 (0.81-1.72); 0.38 | **1.17 (1.10-1.23); <0.01** |
| **Cancer** | **2.13 (1.53-2.98); <0.01** | **3.29 (3.16-3.43); <0.01** |
| **Thyroid Disorders** | **1.71 (1.12-2.60);0.01** | **1.16 (1.08-1.25); <0.01** |
| Prostate Disorders | 1.04 (0.59-1.84);0.87 | 0.96 (0.86-1.07); 0.50 |
| Psoriasis/Eczema | 0.90 (0.44-1.82); 0.77 | 1.08 (0.99-1.17); 0.07 |
| Connective Tissue Disorders* | 1.72 (0.91-3.26);0.09 | **1.63 (1.49-1.78); <0.01** |
| **COPD** | **3.31 (2.14-5.11); <0.01** | **2.70 (2.52-2.90); <0.01** |
| Irritable Bowel syndrome | 1.13 (0.46-2.76);0.77 | **0.80 (0.70-0.91); <0.01** |
| **Osteoporosis** | **3.13 (1.63-6.01);<0.01** | **1.71 (1.54-1.90); <0.01** |
| Diverticular Disease | 0.22 (0.03-1.58);0.13 | 1.11 (0.96-1.29); 0.13 |
| Migraine | 0.89 (0.28-2.79);0.84 | **0.83 (0.74-0.94); <0.01** |
| Glaucoma | 1.63 (0.76-3.46);0.20 | **1.33 (1.17-1.52); <0.01** |
| Anxiety | 1.51 (0.62-3.68); 0.35 | **1.16 (1.03-1.31); 0.01** |

Legend: CI= Confidence Intervals CHD=Coronary heart disease; PVD=peripheral vascular disease; TIA=Transient ischaemic attack; Depressive symptoms=Patient Health Questionnaire-PHQ-2≥2; COPD=Chronic obstructive pulmonary disease; chronic pain symptoms= pain present for more than three months

Painful conditions= back pain, joint pain, headaches (not migraine), sciatica, plantar fasciitis, carpal tunnel syndrome, fibromyalgia, arthritis, shingles, disc problem, prolapsed disc/slipped disc, spine arthritis/spondylitis, ankylosing spondylitis, back problem, osteoarthritis, gout, cervical spondylosis, trigeminal neuralgia, disc degeneration, trapped nerve/compressed nerve.

Connective tissue disorders=myositis/myopathy, systemic lupus erythematosus, Sjogren’s syndrome/sicca syndrome, dermatopolymyositis, dermatomyositis, polymyositis, scleroderma, systemic sclerosis, rheumatoid arthritis, psoriatic arthropathy, polymyalgia rheumatica, malabsorption syndrome/coeliac disease

*Participants with AF N=3651 (97 excluded due to missing values); Results adjusted for age, gender, socioeconomic, smoking and anti-coagulation status.

**Participants without AF N=4989986 (5260 excluded due to missing values); Results adjusted for age, gender, socioeconomic and smoking status.

**Sensitivity Analysis (Results with age as a continuous measure in regression models)**

**Table S3 Title: Relationship of multimorbidity with all-cause mortality in participants with and without self-reported AF using multivariate Cox’s proportional hazards regression analysis**

| Predictor variables | | Hazard Ratios with 95% CI | p-value | Regression coefficients |
| --- | --- | --- | --- | --- |
| Age (as continuous variable) | | 1.08 (1.07-1.08) | <0.001 | 0.077 |
| Sex-male (against female as reference) | | 1.70 (1.65 to 1.76) | <0.001 | 0.53 |
| Townsend Score categories | Category 1-least deprived (reference) | 1 |  |  |
|  | Category 2 | 1.00 (0.95 to 1.06) | 0.78 | 0.007 |
|  | Category 3 | 1.11 (1.05 to 1.17) | <0.001 | 0.10 |
|  | Category 4 | 1.26 (1.20 to 1.33) | <0.001 | 0.23 |
|  | Category 5- most deprived | 1.62 (1.54 to 1.70) | <0.001 | 0.48 |
| Smoking-current/previous (vs. never smoked as reference) | | 1.57 (1.51 to 1.62) | <0.001 | 0.45 |
| Participants classified into different groups based on presence of AF and the number of long term conditions | Participants without any long term conditions-LTCs (reference) | 1 |  |  |
|  | AF present and no other LTCs | 1.63 (1.10 to 2.40) | <0.001 | 0.48 |
|  | AF absent and 1-3 LTCs | 1.77 (1.69 to 1.86) | <0.001 | 0.57 |
|  | AF present and 1-3 other LTCs | 2.43 (2.07 to 2.86) | <0.001 | 0.89 |
|  | AF absent and 4 or more LTCs | 3.38 (3.18 to 3.60) | <0.001 | 1.21 |
|  | AF present and 4 or more other LTCs | 5.30 (4.08 to 6.88) | <0.001 | 1.66 |
| Concordance (measure of model performance)= 0.72 | | | | |

Legend: N=502637 UK Biobank participants (5261 excluded due to missing values); number of deaths=14206; AF=Atrial Fibrillation; outcome= all-cause mortality at 7 years; CI=Confidence Intervals

**Table S4 Title: Relationship between presence of cardiometabolic and non cardiometabolic comorbidity and all-cause mortality in AF participants using multivariate Cox’s proportional hazards model**

| Predictor variables | | Hazard Ratios with 95% CI | p-value | Regression coefficients |
| --- | --- | --- | --- | --- |
| Age (as continuous variable) | | 1.05 (1.02 to 1.08) | <0.001 | 0.05 |
| Sex-male (against female as reference) | | 2.20 (1.58 to 3.06) | <0.001 | 0.78 |
| Townsend Score Categories | Category 1-least deprived (reference) | 1 |  |  |
|  | Category 2 | 1.45 (0.92 to 2.28) | 0.10 | 0.37 |
|  | Category 3 | 1.90 (1.23 to 2.93) | 0.003 | 0.64 |
|  | Category 4 | 1.79 (1.15 to 2.79) | 0.009 | 0.58 |
|  | Category 5- most deprived | 2.09 (1.34 to 3.25) | <0.001 | 0.74 |
| Smoking-current/previous (against never smoked as reference) | | 1.24 (0.95 to 1.62) | 0.10 | 0.21 |
| Anti-coagulation Status-on warfarin (against not on anti-coagulants as reference) | | 1.26 (0.97 to 1.65) | 0.08 | 0.23 |
| Presence of at least one other cardiometabolic condition (against no comorbid cardiometabolic condition as reference) | | 1.78 (1.33 to 2.38) | <0.001 | 0.57 |
| Presence of at least one other non cardiometabolic condition (against no comorbid non-cardiometabolic condition) | | 1.44 (1.09 to 1.89) | 0.008 | 0.36 |
| Concordance (measure of model performance)= 0.68 | | | | |

Legend N=3651 participants with AF (107 excluded due to missing values); number of deaths=241; AF=Atrial Fibrillation; outcome=all-cause mortality at 7 years; CI=Confidence Interval
