# Supplementary material for: Multimorbidity and co-morbidity in atrial fibrillation and effects on survival: findings from UK Biobank cohort
Source: Europace. 2017 Nov 2;20(FI 3):f329–36. doi: 10.1093/europace/eux322 (PMC6277149; doi:10.1093/europace/eux322)
Supplement: Supplementary Material. [file eux322_supplementarymaterial.docx]

Supplementary Material

**Table S1 List of self-reported long-term conditions considered for multimorbidity count**

**Table S2 Hazard Ratio for presence of different cardiometabolic and non-cardiometabolic conditions and all-cause mortality in participants with and without AF**

**Sensitivity Analysis (Results with age as a continuous measure in regression models)**

**Table S3 Title: Relationship of multimorbidity with all-cause mortality in participants with and without self-reported AF using multivariate Cox’s proportional hazards regression analysis**

**Table S4 Title: Relationship between presence of cardiometabolic and non cardiometabolic comorbidity and all-cause mortality in AF participants using multivariate Cox’s proportional hazards model**
